# Epidemiological and clinical differences between sexes and pathogens in a three-year surveillance of acute infectious gastroenteritis in Shanghai

**DOI:** 10.1038/s41598-019-46480-6

**Published:** 2019-07-10

**Authors:** Lingfei Luo, Yiqin Gu, Xiaoguang Wang, Yinghua Zhang, Longwen Zhan, Jiqian Liu, Hongjing Yan, Yun Liu, Shanshan Zhen, Xiuhua Chen, Rui Tong, Chiping Song, Yingying He

**Affiliations:** The Center for Disease Control and Prevention of Minhang District, Minhang District, 965 Zhongyi Avenue, Shanghai, 201101 P.R. China

**Keywords:** Clinical microbiology, Epidemiology

## Abstract

Acute infectious gastroenteritis cases in Shanghai, reported over three years, were analyzed. Pathogens were identified in 1031 patients; of these, 725 and 306 were bacterial and viral cases, respectively. *Vibrio parahemolyticus* and *Salmonella* were the dominant bacteria, and *Caliciviridae* and *Reoviridae* were the dominant viral families in the local area. The acute gastroenteritis epidemic peaks appeared in August and January, which represented the active peak periods of bacteria and viruses, respectively. Logistic regression analyses with sex stratification showed that abdominal pain, fever and ingestion of unsafe food at restaurants were independent factors more frequently associated with bacterial gastroenteritis irrespective of sex; red cell-positive fecal matter was associated with bacterial gastroenteritis with an odds ratio (OR) of 3.28 only in males; and white blood cell count was associated with bacterial gastroenteritis with an OR of 1.02 only in females. Pathogen stratification showed that age, vomiting and red cell-positive fecal matter were associated with males with ORs of 0.99, 0.61 and 1.71, respectively, in bacterial gastroenteritis; and the migrant ratio was higher in males with an OR of 2.29 only in viral gastroenteritis. In conclusion, although bacterial and viral gastroenteritis shared many features, epidemiological and clinical factors differed between sexes and pathogens.

## Introduction

Acute gastroenteritis is a common disease that is usually caused by viral, bacterial or parasitic infections in the digestive tract^1^. Outbreaks and sporadic cases of gastroenteritis occur throughout the year and pose a major public health burden worldwide^1–3^. Most causes of acute gastroenteritis are foodborne or waterborne due to the contamination of a pathogen toxin and/or toxic microorganism^4^. Of the infectious causes of acute gastroenteritis, it is estimated that 50–70%, 15–20% and 10–15% are caused by viral, bacterial and parasitic infections, respectively^1^. In general, acute viral gastroenteritis peaks in the autumn and winter seasons, and the peak of acute bacterial gastroenteritis appears in summer^1–3^.

The main clinical manifestations of acute gastroenteritis are sudden-onset vomiting or diarrhea with or without accompanying nausea, fever, or abdominal pain^4–6^. It is hard for clinicians to differentiate viral gastroenteritis from gastroenteritis caused by bacterial agents on the basis of clinical manifestations alone^7^. Although viral gastroenteritis manifests abruptly with vomiting and watery diarrhea, often accompanied by low-grade fever and abdominal cramps, these clinical manifestations are always shared by bacterial gastroenteritis^6–8^. Thus, further evaluation of differences in sex, infection source, transmission route, symptoms as well as clinical laboratory tests between viral and bacterial gastroenteritis has clinical significance. In addition, Shanghai is an international metropolis that had 24.2 million residents and 320 million tourists in the year of 2017, and high population density is a major factor promoting acute gastroenteritis epidemics^2,3,9–12^. So far, long-term acute gastroenteritis surveillance data with a large sample size from Shanghai are still scarce. Furthermore, the pathogenic spectrum of acute infectious gastroenteritis prevalent in the local area remains unclear.

In this report, acute gastroenteritis surveillance data collected over a three-year span from two sentinel hospitals in Shanghai are presented. The epidemiological and clinical differences between viral and bacterial gastroenteritis as well as between the sexes and the spectrum of acute gastroenteritis-related viruses and bacteria in the local area were evaluated.

## Materials and Methods

### Ethics issues

This study was conducted in accordance with the World Medical Association Declaration of Helsinki and was approved by the Internal Review Board of the Center for Disease Control and Prevention of Minhang District, Shanghai. Written informed consent was obtained according to the guidelines of the National Ethics Regulation Committee.

### Study design and sampling

This report focused on individuals with suspected acute viral and bacterial gastroenteritis. Acute gastroenteritis was defined as sudden-onset vomiting or diarrhea with or without accompanying nausea, fever, or abdominal pain. Acute diarrhea refers to an individual who presents with an abrupt onset of 3 or more loose or liquid stools above baseline in a 24 h period^6^.

We analyzed the annual registered data from 2014, 2015 and 2016 from two sentinel hospitals in the Minhang District of Shanghai, the 5th People’s Hospital of Shanghai and the Qibao Community Health Service Center. According to the Comprehensive Monitoring Scheme for Diarrhea in Shanghai, age, sex, profession, initial symptoms, symptoms, history of ingesting unsafe foods (According to the definition from CODEX ALIMENTARIUS COMMISSION PROCEDURAL MANUAL released by the Food and Agriculture Organization of the United Nations^13,14^, unsafe food was defined as “biological, chemical or physical agent in, or condition of, food with the potential to cause an adverse health effect”. Foodborne events were investigated by a foodborne disease questionnaire recommended by the WHO^15^, which includes sections of demographic, clinical and exposure information; food history; source of food; and eating site^15^. In addition, FIVE KEYS TO SAFER FOOD MANUAL proposed by WHO^16^, i.e., keep clean, separate raw and cooked, cook thoroughly, keep food at safe temperatures, and use safe water and raw materials; were also included in the questionnaire survey), history of drinking unsafe water (expired bottled water or raw water, such as well water or river water^17^), contact with diarrheal patients, travel 5 days before the onset of diarrhea, and administration of antibiotics were recorded. Watery diarrhea, bloody diarrhea, dehydration, fever, heart rate, blood pressure, routine blood examination, and a stool test were checked by the initial clinicians or performed in the clinical laboratory of the hospital.

All individuals with suspected infectious acute gastroenteritis older than 18 years and registered in the years 2014, 2015 or 2016 by the two sentinel hospitals were included in this report; individuals with known poisoning (e.g., mushroom poisoning), diarrhea for >7–10 days (considered chronic disease), bilious emesis, acute surgical abdomen, and bloody diarrhea were excluded. Based on our long-term survey data, acute gastroenteritis related to enteric parasites (such as Giardia, Cryptosporidium and *Entamoeba histolytica*) is now rare in adults living in Shanghai; thus, we did not perform detection for enteric parasites.

For pathogen detection, in order to reduce the workload and ensure the accuracy and effectiveness of pathogen detection, random sampling to collect stool or vomit samples is always adopted in an acute gastroenteritis survey. The hospital with more patients performed detection with a 1:20 sampling rate (the 5th People’s Hospital of Shanghai), while the hospital with fewer patients performed detection with a 1:5 sampling rate (Qibao Community Health Service Center).

### Isolation and identification of bacterial pathogens

Bacterial detection is a vital part of the acute gastroenteritis control and prevention strategy established by the local health administration department. Bacterial identification is the systematic work of a multisectoral collaboration in China’s Centers for Disease Control and Prevention, and it is difficult to detail all processes in this report. Briefly, detection was carried out via classic bacterial isolation, culture, and identification in combination with molecular diagnostic strategies^2^. In addition, bacterial samples were subjected to VITEK® 2 Compact (bioMérieux, France) for simultaneous microbial identification. The above technologies covered the most common diarrhea-related bacteria, including *Vibrio cholerae, Salmonella, Shigella, Vibrio parahemolyticus, Yersinia enterocolitica, Campylobacter jejuni, Enterotoxigenic Escherichia coli (ETEC)*, *Enteropathogenic E. coli (EPEC)* and others^2^. To learn the pathotypes of diarrheagenic *E. coli*, suspected *E. coli* colonies on SMAC or MacConkey plates were further analyzed by PCR using primers targeting the genes *eae, bfp, stx1, stx2, ipaH, pCDV, eltA* and *estA*. A first PCR detection was performed with *stx1/stx2* and *eae* primers focusing on the identification of STEC or EPEC. Positive *eae* and negative *stx1/stx2* samples were then examined by PCR with *bfp* primers to differentiate tEPEC from aEPEC. Negative *eae* and *stx1/stx2* samples were further screened by PCR using pCVD432 primers for plasmidic EAEC sequences, *ipaH* primers for detecting invasion plasmid antigen genes of EIEC, and detection of the *eltA* and *estA* genes of ETEC labile and stable enterotoxins^18,19^. The diagnosis of epidemic dysentery and cholera was performed according to the Laboratory Methods for the Diagnosis of Epidemic Dysentery and Cholera recommended by the U.S. Centers for Disease Control and Prevention and the World Health Organization^5^.

Laboratory standard operating procedures for detection were performed according to the PRACTICAL GUIDANCE FOR CLINICAL MICROBIOLOGY-Laboratory Diagnosis of Bacterial Gastroenteritis and the Final Report and Executive Summaries from the AOAC International Presidential Task Force on Best Practices in Microbiological Methodology^8^.

### Detection of viral pathogens

Viral detection is another vital part of the acute gastroenteritis control and prevention strategy established by the local health administration department. Gastroenteritis virus detection was performed according to methods reported by other peers^2,10,12,20,21^. Briefly, fecal specimens were prepared as 10% (w/v) suspensions in distilled water and then centrifuged for 10 min at 10000 *g* in a 1.5 ml collection tube (Biovisualab, Shanghai, China) to remove debris. Viral DNA and viral RNA were extracted from the suspensions using a QIAamp DNA Mini Kit and a QIAamp Viral RNA Mini Kit (Qiagen, The Netherlands), according to the manufacturer’s instructions. The virus panel was established with routine diarrhea surveillance data that were accumulated by local Centers for Disease Control and Prevention and by reference to information from peers^20–23^. Rotavirus, norovirus, enteric adenovirus, astrovirus, sapovirus, mimiviruses, aichivirus, bocavirus, parechovirus, cytomegalovirus, hepatitis A, coronaviruses, picornaviruses, toroviruses, and other enteroviruses were detected by polymerase chain reaction (PCR) or reverse transcription PCR using primer sets, as reported previously^2,10,12,20–23^.

### Statistical analysis

Continuous variables are presented as the means ± standard deviations (SDs); differences between groups were evaluated using the Mann-Whitney *U*-test for independent samples. Categorical variables are presented as frequencies (percentage); differences in frequencies were evaluated using the Chi-square test or Fisher’s exact probability test. To identify independent factors and to distinguish factors associated with pathogen or sex, logistic regression analyses were applied in 1031 subjects with known pathogens. To identify factors that tended to be associated with pathogens, the dependent variables were defined as viral case = 0 and bacterial case = 1, and the multivariable logistic regression analyses were performed by sex. To identify factors that tended to be associated with sex, the dependent variables were defined as female = 0 and male = 1, and the multivariable logistic regression analyses were performed by viral and bacterial gastroenteritis. Potential independent variables were selected by univariate analyses; factors with P < 0.10 were introduced into the starting model of the multivariable logistic regression analyses and then eliminated manually using the backward step-by-step approach, depending on the largest p-value. All analyses were performed using SPSS software for Windows (ver. 18.0; SPSS Inc., Chicago, IL, USA), and the significance level (alpha) was set at 0.05.

## Results

### General information from three-year-round surveillance

This report included annual cases from three full years of acute infectious gastroenteritis that occurred in two sentinel hospitals of Shanghai from 2014 to 2016. In total, 11243 subjects, 5857 males and 5386 females, were involved.

As shown in Table 1, the average age of patients was older among females, and the ratio of local residents was significantly higher in the female group. Regarding the symptoms associated with acute gastroenteritis, the percentages of subjects with nausea, vomiting, watery stools and abdominal pain were significantly higher in the female group than in the male group. The rate of patients with fever was significantly higher in males (9.4%) than in females (7.1%). No significant difference in vomiting frequency, duration of vomiting, diarrhea frequency, duration of diarrhea, average body temperature or rate of dehydration existed between the sexes. Since the number of patients with dehydration was low in both sexes, differences in blood pressure may be an inherent sex difference rather than a pathological feature associated with acute gastroenteritis.

**Table 1:** General information by sex.

	Male	Female	*P*
***Demographic characteristics***			
Number (%)	5857 (52.1)	5386 (47.9)	/
Age	39.9 ± 17.2	43.3 ± 17.9	<*0.001*
Local residents (%)	4844 (82.7)	4758 (88.3)	<*0.001*
Migrants and floating population (%)	1013 (17.3)	628 (11.7)	
***Symptoms***			
Nausea (%)	1978 (33.8)	2287 (42.5)	<*0.001*
Vomiting (%)	1138 (19.4)	1598 (29.7)	<*0.001*
Vomiting frequency/day	2.3 ± 1.8	2.5 ± 1.9	*0.569*
Duration of vomiting, day	1.0 ± 0.3	1.0 ± 0.2	*0.309*
Diarrhea (%)	5841 (99.7)	5379 (99.9)	*0.093*
Watery stools (%)	5011 (85.6)	4717 (87.6)	*0.002*
Diarrhea frequency/day	5.8 ± 2.9	6.0 ± 3.0	*0.388*
Duration of diarrhea, day	1.3 ± 1.2	1.3 ± 2.3	*0.309*
Abdominal pain (%)	2295 (39.2)	2530 (47.0)	<*0.001*
Fever (%)	549 (9.4)	382 (7.1)	<*0.001*
Average body temperature, °C	38.1 ± 0.5	38.0 ± 0.4	*0.288*
Mild dehydration (%)	166 (2.8)	165 (3.1)	*0.471*
Moderate dehydration (%)	1 (0.02)	0 (0.0)	/
Heart rate	77.4 ± 5.7	77.1 ± 6.2	*0.669*
Systolic blood pressure, mmHg	114.7 ± 14.0	109.4 ± 17.4	*0.002*
Diastolic blood pressure, mmHg	74.9 ± 8.5	70.9 ± 9.0	<*0.001*
Symptoms of neurotoxicity	0 (0.0)	0 (0.0)	/
***Epidemiological factors***			
Unsafe foods (%)	3696 (63.1)	3449 (64.0)	*0.306*
Situs: restaurants (%)	930 (25.2)	678 (19.7)	<*0.001*
Situs: home (%)	2438 (66.0)	2531 (73.4)	<*0.001*
Situs: delivery foods (%)	254 (6.9)	173 (5.0)	*0.001*
Unsafe water (%)	1 (0.02)	1 (0.02)	/
Exposure to diarrhea within 5 days (%)	17 (0.3)	6 (0.1)	/
Travel within one week (%)	5 (0.1)	0 (0.0)	/
***Clinical laboratory tests***			
Fecal leukocyte positive (%)	1250/4250 (29.4)	1110/3891 (28.5)	*0.380*
Fecal red cell positive (%)	458/4149 (11.0)	354/3784 (9.4)	*0.013*
Blood routine test (%)	5025 (85.8)	4911 (91.2)	/
White blood cell count, x10^9/L	73.0 ± 13.8	74.5 ± 13.7	*0.394*

Continuous variables are presented as the means ± standard deviations (SDs); differences between groups were evaluated using the Mann-Whitney *U*-test for independent samples. Categorical variables are presented as frequencies (percentages); differences in frequencies were evaluated using the Chi-square test or Fisher’s exact probability test.

The epidemiological questionnaire showed that 63.1% and 64.0% of male and female patients, respectively, had a history of the possible ingestion of unsafe foods. The foodborne source of males more frequently tended to be from restaurants and delivery foods, while the foodborne source of females more frequently tended to be from home (Table 1). The rates of drinking unsafe water, exposure to patients with diarrhea within 5 days and travel within one week were very low in both sexes (Table 1). Clinical laboratory tests showed that the fecal red cell-positive rate was significantly lower in females (9.4%) than in males (11.0%) (Table 1).

In conclusion, age, symptoms, epidemiological factors and clinical laboratory tests differed between sexes in patients with acute gastroenteritis.

### Annual dynamic characteristics of the acute gastroenteritis epidemic

**Figure 1:**
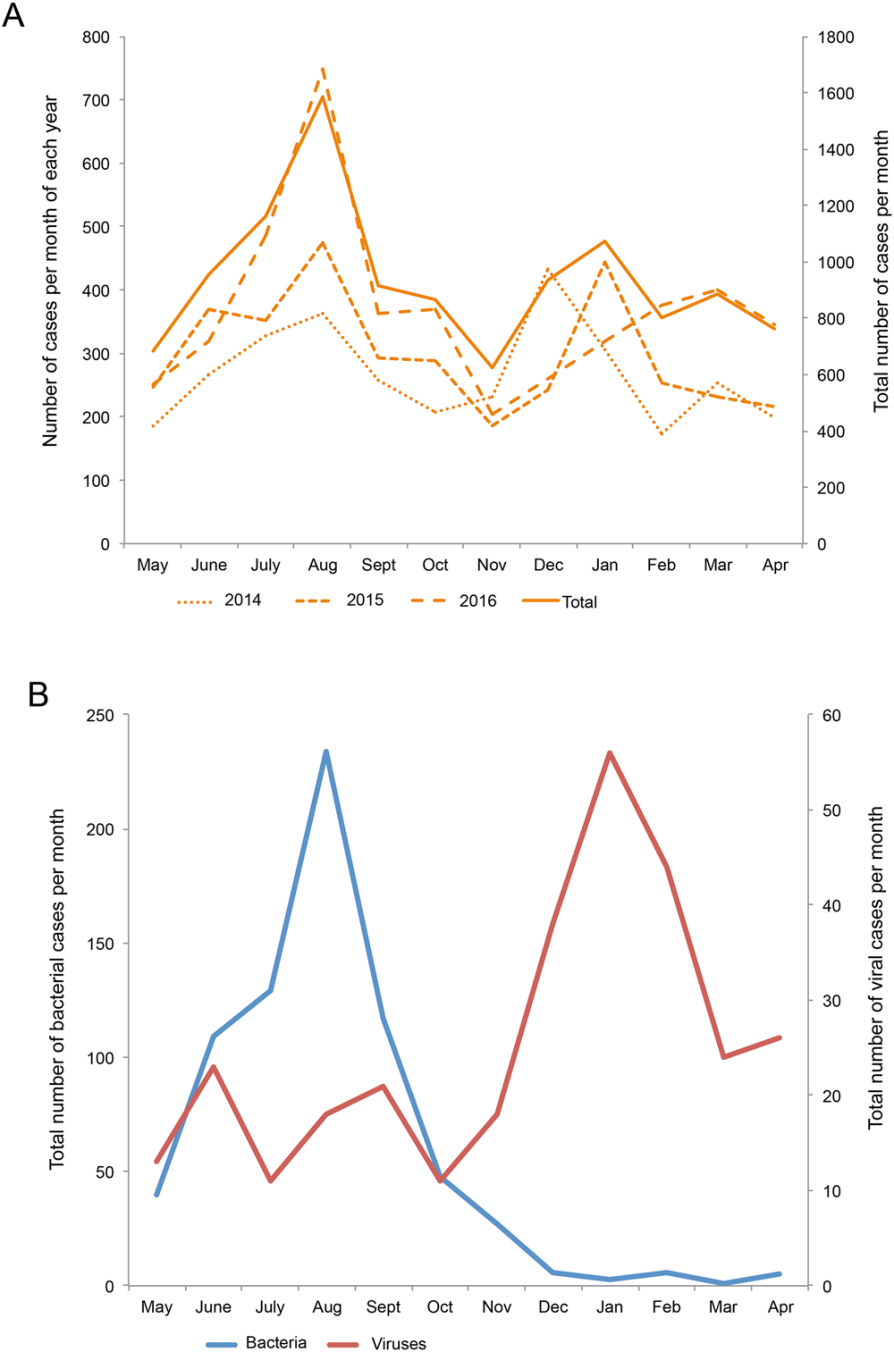
Seasonal distribution of infectious diarrhea. (**A**) Three years of annual incidence data of acute infectious gastroenteritis from two sentinel hospitals were aligned by month. To better present the two peaks of the disease epidemics, the time axis was arranged from May to December and from the following January to April. (**B**) Subjects with identified pathogens were also aligned by month. To better present the two peaks of the viral and bacterial gastroenteritis epidemics, the time axis was arranged from May to December and from the following January to April.

To understand the seasonal epidemic characteristics of acute infectious gastroenteritis, all cases were aligned by month. As shown in Fig. 1A, all annual acute gastroenteritis epidemics, including 2014, 2015, 2016 and merged data, displayed two peaks in the summer and winter. To learn if the epidemic peaks were associated with pathogen differences, all identified viral and bacterial cases were aligned again by month. As shown in Fig. 1B, cases infected by bacteria peaked in August, and cases infected by viruses peaked in January.

### Pathogenic spectrum

**Figure 2:**
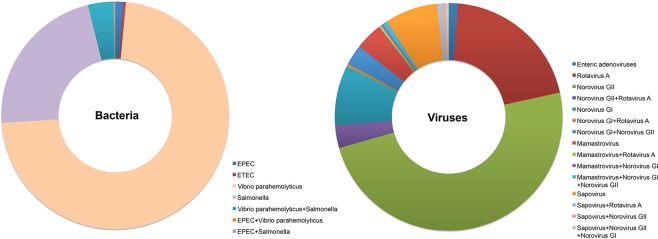
Pathogenic spectrum of acute gastroenteritis. Identified bacteria and viruses were presented as ratios, and the proportions of bacterial or viral coinfections were calculated separately and were not included in the individual proportion of each pathogen.

Pathogens were successfully identified in 1031 patients, and 725 and 306 subjects were infected by bacteria and viruses, respectively. Of the identified cases of bacterial acute gastroenteritis, 72.4%, 22.2%, 1.1% and 0.4% were infected by *Vibrio parahemolyticus, Salmonella*, EPEC and ETEC alone, respectively; 3.6%, 0.1% and 0.1% were coinfected by *Vibrio parahemolyticus* and *Salmonella*, EPEC and *Vibrio parahemolyticus* and EPEC and *Salmonella*, respectively (Fig. 2).

Of the cases with identified viral acute gastroenteritis, 49.0%, 20.3%, 8.5%, 7.5%, 3.9% and 1.3% were infected by norovirus GII, rotavirus A, norovirus GI, sappovirus, mamastrovirus and enteric adenoviruses alone, respectively. In all, 3.3%; 2.9%; 0.7%; 0.7%; 0.7%; 0.3%; 0.3%; 0.3% and 0.3% of subjects were coinfected by norovirus GII and rotavirus A; norovirus GI and norovirus GII; mamastrovirus, norovirus GI and norovirus GII; sapovirus and rotavirus A; sapovirus and norovirus GII; norovirus GI and rotavirus A; mamastrovirus and rotavirus A; mamastrovirus and norovirus GI; and sapovirus and norovirus GII and norovirus GI, respectively (Fig. 2).

### Differences between viral and bacterial gastroenteritis by sex

The difference between the sexes shown in Table 1 does not taken into account the possible role of pathogens. Therefore, the clinical and epidemiological characteristics of bacterial and viral cases were further compared by sex.

Of males, as shown in Table 2, 366 and 177 subjects were identified as having bacterial and viral gastroenteritis, respectively. The rates of nausea, watery stools, abdominal pain, fever, ingesting possible unsafe foods, ingesting unsafe foods at restaurants, leukocyte- and red-cell-positive fecal matter and the average vomiting frequency, body temperature and white blood cell count variables were significantly higher in the bacterial group than in the viral group; conversely, the average vomiting duration, heart rate and diastolic pressure and the rate of ingesting unsafe foods at home variables were significantly higher in the virus group than in the bacterial group. No significant difference in the age, percentage of local residents, vomiting rate, diarrhea frequency, duration of diarrhea, dehydration rate, systolic blood pressure, or rate of ingesting delivery food variables was observed between the viral and bacterial gastroenteritis groups (Table 2, left panel).

**Table 2:** Clinical and epidemiological differences between viral and bacterial gastroenteritis by sex.

	Males			Females		
	Bacteria	Viruses	*P*	Bacteria	Viruses	*P*
***Demographic characteristics***						
Number	366	177	*/*	359	129	*/*
Age	39.4 ± 15.5	40.9 ± 16.6	*0.146*	42.0 ± 17.1	44.1 ± 17.2	*0.110*
Local residents (%)	296 (80.9)	134 (75.7)	*0.165*	309 (86.1)	114 (88.4)	*0.512*
Migrants and floating population (%)	70 (19.1)	43 (24.3)		50 (13.9)	15 (11.6)	
***Symptoms***						
Nausea (%)	155 (42.3)	61 (34.5)	*0.047*	192 (53.5)	51 (39.5)	*0.007*
Vomiting (%)	99 (27.0)	43 (24.3)	*0.282*	147 (40.9)	39 (30.2)	*0.020*
Vomiting frequency/day	2.8 ± 2.1	2.1 ± 1.8	*0.035*	3.0 ± 2.3	3.2 ± 2.2	*0.335*
Duration of vomiting, day	1.0 ± 0.2	1.1 ± 0.4	*0.047*	1.0 ± 0.1	1.1 ± 0.4	*0.015*
Diarrhea (%)	366 (100.0)	177 (100.0)	*/*	359 (100.0)	129 (100.0)	*/*
Watery stools (%)	326 (89.1)	145 (81.9)	*0.016*	318 (88.6)	113 (87.6)	*0.438*
Diarrhea frequency/day	6.3 ± 3.0	6.1 ± 3.6	*0.263*	6.2 ± 2.9	6.1 ± 2.8	*0.275*
Duration of diarrhea, day	1.3 ± 1.2	1.3 ± 0.8	*0.223*	1.3 ± 0.9	1.2 ± 0.7	*0.120*
Abdominal pain (%)	224 (65.1)	64 (36.2)	<*0.001*	248 (69.1)	50 (38.8)	<*0.001*
Fever (%)	69 (18.9)	19 (10.7)	*0.010*	56 (15.6)	7 (5.4)	*0.003*
Average body temperature, °C	38.2 ± 0.5	37.9 ± 0.4	*0.005*	38.1 ± 0.4	37.9 ± 0.3	*0.107*
Mild dehydration (%)	22 (6.0)	5 (2.8)	*0.110*	14 (3.9)	2 (1.6)	*0.159*
Moderate dehydration (%)	0 (0.0)	0 (0.0)	*/*	0 (0.0)	0 (0.0)	*/*
Heart rate	75.7 ± 5.5	80.8 ± 1.8	*0.030*	77.0 ± 5.5	75.0 ± 7.1	*0.322*
Systolic blood pressure, mmHg	116.8 ± 11.2	121.0 ± 15.2	*0.247*	111.4 ± 18.1	110.0 ± 10.0	*0.458*
Diastolic blood pressure, mmHg	73.2 ± 7.8	81.0 ± 2.2	*0.021*	71.1 ± 9.6	75.0 ± 7.1	*0.296*
Symptoms of neurotoxicity	0 (0.0)	0 (0.0)	*/*	0 (0.0)	0 (0.0)	*/*
***Possible etiologic factors***						
Unsafe foods (%)	363 (99.2)	166 (93.8)	<*0.001*	356 (99.2)	125 (96.9)	*0.083*
Situs: restaurants (%)	148 (40.8)	48 (28.9)	*0.009*	123 (34.6)	27 (21.6)	*0.004*
Situs: home (%)	211 (58.1)	117 (70.5)	*0.007*	230 (64.6)	98 (78.4)	*0.003*
Situs: delivery foods (%)	4 (1.1)	1 (0.6)	*0.499*	6 (1.7)	0 (0.0)	*/*
Unsafe water (%)	1 (0.3)	0 (0.0)	*/*	0 (0.0)	0 (0.0)	*/*
Exposure to diarrhea within 5 days (%)	2 (0.6)	2 (1.1)	*/*	2 (0.6)	0 (0.0)	*/*
Travel within one week (%)	0 (0.0)	0 (0.0)	*/*	0 (0.0)	0 (0.0)	*/*
***Clinical laboratory tests***						
Fecal leukocyte positive (%)	122/323 (37.8)	42/156 (26.9)	*0.019*	103/297 (34.7)	26/118 (22.0)	*0.008*
Fecal red cell positive (%)	67/314 (21.3)	10/152 (6.6)	<*0.001*	46/293 (15.7)	5/115 (4.3)	*0.001*
White blood cell count, x10^9/L	78.2 ± 12.5	75.6 ± 12.8	*0.014*	79.5 ± 11.6	74.5 ± 15.0	*0.001*

Continuous variables are presented as the means ± standard deviations (SDs); difference between groups were evaluated using the Mann-Whitney U-test for independent samples. Categorical variables are presented as frequencies (percentages); differences in frequencies were evaluated using the Chi-square test or Fisher’s exact probability test.

Of females, 359 and 129 subjects were identified as having bacterial and viral gastroenteritis, respectively. The rates of nausea, vomiting, abdominal pain, fever, ingesting unsafe foods at restaurants, leukocyte- and red-cell-positive fecal matter and the white blood cell count variables were significantly higher in the bacterial group than in the viral group; conversely, the average vomiting duration was significantly longer and the rate of ingesting unsafe foods at home was significantly higher in the viral group than in the bacterial group. No significant differences in the age, percentage of local residents, frequency of vomiting, frequency of watery stools, frequency of diarrhea, duration of diarrhea, rate dehydration, heart rate, blood pressure, or rate of ingesting possible unsafe food variables was observed between the viral and bacterial gastroenteritis groups (Table 2, right panel).

### Independent factors differentially associated with pathogen by sex

Bacterial gastroenteritis shares many clinical manifestations and epidemiological features with viral gastroenteritis^6–8^. Although the above stratified analyses showed differences between sexes and pathogens, such analyses could neither identify independent factors nor quantify the associations. Thus, logistic regression analyses were adopted to distinguish the associations.

Among males, univariate analyses showed that nausea, vomiting frequency, watery stools, abdominal pain, fever, ingesting unsafe food at restaurants, fecal leukocyte-positive, fecal red cell-positive and white blood cell count were potential independent factors that were differentially associated with viral and bacterial gastroenteritis. Multivariable logistic regression analyses revealed that only abdominal pain, fever, fecal red cell-positive and ingesting unsafe food at restaurants were independent factors that more frequently occurred in bacterial gastroenteritis than viral gastroenteritis with ORs of 2.61, 2.15, 3.28 and 1.59, respectively (Table 3).

**Table 3:** Independent factors associated with bacterial gastroenteritis by sex.

Variable	Female (N = 488)			Male (N = 543)		
	OR	95% CI	*P*	OR	Lower 95% CI	*P*
Abdominal pain, yes vs. no	3.05	1.94~4.79	<*0.001*	2.61	1.74~3.92	<*0.001*
Fever, yes vs. no	3.54	1.34~9.40	*0.011*	2.15	1.13~4.08	*0.019*
Fecal red cell positive, yes vs. no	/	/	*/*	3.28	1.56~6.90	*0.002*
White blood cell count, x10^9/L	1.02	1.01~1.04	*0.011*	/	/	*/*
Unsafe food ingesting situs, restaurant vs. home	1.72	1.03~2.89	*0.039*	1.59	1.04~2.42	*0.032*

OR, odds ratio; 95% CI, 95% confidence interval. OR for white blood cell count = OR for an increase of 1 unit.

Among females, nausea, vomiting, duration of vomiting, abdominal pain, fever, ingesting unsafe food at restaurants, fecal leukocyte-positive, fecal red cell-positive and white blood cell count were potential independent variables that were differentially associated with viral and bacterial gastroenteritis. Further multivariable logistic regression analyses revealed that abdominal pain, fever, and ingesting unsafe food at restaurants were independent factors that more frequently occurred in bacterial gastroenteritis with ORs of 3.05, 3.54 and 1.72, respectively (Table 3). White blood cell count was also an independent factor and higher in bacterial gastroenteritis than in viral cases (Table 3).

### Independent factors differentially associated with sex by pathogen

To identify the independent factors of acute infectious gastroenteritis that were differentially associated with sex by pathogen, logistic regression analyses were applied to 725 bacterial and 306 viral cases. Initial univariate analyses showed that age, domiciliary register and vomiting frequency and age, domiciliary register, nausea, abdominal pain, vomiting and fecal red cell-positive were potential independent variables in associated with sex in the viral and bacterial groups, respectively. Further multivariable logistic regression analyses using the above candidate variables revealed that, in bacterial gastroenteritis, an increase of 1 year old resulted in a decreased male/female (ratio) with an OR of 0.99 (Table 4), vomiting occurred less frequently in males than in females with an OR of 0.61, and red cell-positive fecal matter occurred more frequently in males than in females with an OR of 1.71 (Table 4). In the viral group, only domiciliary register remained an independent factor associated with males with an OR of 2.29 (migrant vs. local resident) (Table 4).

**Table 4:** Independent factors associated with males by pathogen.

Variable	Bacteria (N = 725)			Viruses (N = 306)		
	OR	95% CI	*P*	OR	Lower 95% CI	*P*
Age, years old	0.99	0.98~1.00	*0.011*	/	/	*/*
Vomiting, yes vs. no	0.61	0.43~0.87	*0.006*	/	/	*/*
Fecal red cell positive, yes vs. no	1.71	1.12~2.61	*0.013*	/	/	*/*
Domiciliary register, migrant vs. local resident	/	/	*/*	2.29	1.20~4.38	*0.012*

OR, odds ratio; 95% CI, 95% confidence interval. OR for age = OR for an increase of 1 year old.

## Discussion

In this report, we collected data across a 3-year period from two sentinel hospitals located in the Minhang District of Shanghai. Our analysis strategy consisted of three steps: first a pooled analysis with 5857 male and 5386 female patients irrespective of pathogens to understand sex differences; second, sex and pathogen stratification analyses with 1031 patients whose pathogens were identified to determine epidemiological and clinical differences between sexes and pathogens; and third, logistic regression analyses to distinguish the factors associated with sex and pathogens.

In the pooled analysis, the percentage of local residents and the rate of ingesting possible unsafe food at home variables were lower in males, and the rate of ingesting possible unsafe food at restaurants was higher in males, which is consistent with the characteristics of male social behavior. The rates of nausea, vomiting, watery stools and abdominal pain were higher, and the rates of fever and red cell-positive fecal matter were lower in females, suggesting a sex difference in symptoms associated with acute gastroenteritis. Although the pooled analysis did not implement pathogen classification, it reflected the features of acute gastroenteritis that clinicians face every day. Next, stratification analyses by sex and pathogen showed that the distributions of clinical and epidemiological factors differed not only between viral and bacterial groups but also between sexes. The results obtained by foregoing two steps guided us to implement sex and pathogen stratification in subsequent logistic regression analyses.

Independent factors differentially associated with pathogens were evaluated by sex stratification; logistic regression analyses revealed that abdominal pain and fever were two common independent symptom factors that more frequently occurred in bacterial gastroenteritis than in viral gastroenteritis, regardless of sex. Clinically, acute infectious gastroenteritis is classified into two pathophysiologic types: noninflammatory and inflammatory. The noninflammatory gastroenteritis is mostly caused by viral infection with milder disease; while, inflammatory gastroenteritis is more severe and always resulted from infection of invasive or with toxin-producing bacteria^1,24–26^. In addition, fever and abdominal pain are two common symptoms in acute gastroenteritis caused by *Salmonella* infection^24^; our data showed that 22.2% identified bacterial cases were *Salmonella* infection (Fig. 2). Thus, higher prevalence of fever and abdominal pain in acute bacterial gastroenteritis were observed. Ingesting unsafe food at restaurants was a common transmission route for both sexes and was more frequently associated with bacterial gastroenteritis; which is consistent with that bacterial infections are more often associated with foodborne transmission^24–26^ and acquired easily at places with high population mobility^27,28^. White blood cell count was higher in bacterial cases only in females with low OR of 1.02, suggesting the relative pathology of infectious acute gastroenteritis differed between sexes. However, testing stool for leukocytes to screen for inflammatory diarrhea has fallen out of favor due to a wide variability in sensitivity and specificity^24,29^.

Independent factors differentially associated with sex were further evaluated in logistic regression analyses with pathogen stratification. In bacterial gastroenteritis, age and vomiting were associated with males with ORs of 0.99 and 0.61, respectively; for ease of understanding, these associations were translated to females, and age and vomiting were associated with females with ORs of 1.01 and 1.64, respectively, meaning that with a 1-year old increase, the female/male ratio will increase 1.01 times, and vomiting is more frequently associated with female patients. The age quartiles of female and male bacterial cases are 27.6, 36.9 and 57.2; and 28.1, 34.9 and 49.9 years old respectively; the 2^nd^ and particularly the 3^rd^ quartiles of female age were older; which is why female/male ratio will increase 1.01 times with a 1-year old increase. Red cell-positive fecal matter remained an independent factor that was more frequently observed in males with OR of 1.71 (Table 4); in addition, the above sex stratification analysis showed that red cell-positive fecal matter was an independent factor that was more frequently observed in bacterial cases only in males with OR of 3.28 (Table 3). These two analysis methods mutually confirmed that red cell-positive fecal matter is common in males with bacterial infections. Bacteria, such as *Vibrio parahemolyticus* and *Salmonella*, predisposing to cause inflammatory infections with bloody stool^24–26^, which could be used to explain why bloody stool is more frequently observed in bacterial infections, but could not interpret gender difference. Since the proportions of *Vibrio parahemolyticus* and *Salmonella* infections were similar between males (49.9% and 52.5%) and females (50.1% and 47.5%), higher bloody stool rate in male cases should not be caused by differences in rates of bacterial infection. Although we could not give a reasonable explanation to partial gender differences; these evidences have clinical significance and will guide translational study to interpret a pathological mechanism. In fact, gender differences exist widely in clinical medicine and have been paid more and more attention in recent years^30^. In viral cases, only domiciliary register remained an independent factor associated with males; the migrant/local resident ratio was 2.29 times higher in males than in females, suggesting the prevalence of viral gastroenteritis is relatively higher in migrant workers.

Some parameters, such as body temperature, heart rate and blood pressure, were not potential independent variables in univariate analyses; this finding is because body temperature was only recorded in subjects with fever, and heart rate and blood pressure were only recorded in patients with dehydration. As to why some variables did not remain independent factors in the final equation, this is partially explained by the high colinearity between variables, for example, the Pearson correlation coefficient between fecal leukocyte-positive and fecal red cell-positive was 0.423 (*P* < 0.001), and the narrow differences between two groups, for example, the percentage of nausea between bacterial and viral cases (42.3% vs. 34.5%) in males and the vomiting frequency between bacterial and viral cases (2.8 ± 2.1 vs. 2.1 ± 1.8 days) in males. However, most of the above differences involved complex pathophysiological principles, and we cannot offer a perfect explanation. Regardless, logistic regression analysis can effectively assess confounders and select independent variables associated with sex and pathogen.

Acute gastroenteritis caused by viral infections was dominated by rotavirus and norovirus^31,32^. Rotavirus always causes severe gastroenteritis in young children^33,34^, while norovirus causes most outbreaks of nonbacterial acute gastroenteritis in all age groups^35^. Recently, some scholars reported that the rotavirus predominance of acute gastroenteritis has been replaced by norovirus due to the wide implementation of rotavirus vaccination^9^. Our data showed that *Caliciviridae* (norovirus GII and GI) and *Reoviridae* (rotavirus A) constituted more than 80% of viral infections. Since a norovirus vaccine is still under development^36^, rotavirus vaccination needs to be implemented in local areas. Bacteria are the second leading cause of acute gastroenteritis; *Shigella, Salmonella, Campylobacter, diarrhoeagenic Escherichia coli, pathogenic Vibrio, Yersinia*, and *Clostridium difficile* are the most commonly reported bacteria correlated with acute gastroenteritis^1,2^. Our data showed that *Vibrio parahemolyticus* and *Salmonella* were the dominant bacteria identified in the local area, and these findings are consistent with those of a previous study with a small sample size^2^.

This report included the following limitations: First, this report only focused on the outpatients of two sentinel hospitals, and all subjects were sporadic adult cases; thus, this study does not represent the infectious gastroenteritis epidemic in schools, although it is known that acute gastroenteritis is highly prevalent in schools and always causes outbreaks^37,38^. Second, based on our sampling rates, pathogens were only identified in 9.2% (1031/11243) of patients, and more effort should be made to improve the representative accuracy of our results. Third, in the stratified and logistic regression analyses, we only considered mixed viral cases and mixed bacterial cases and did not subgroup the subjects by bacterial classification, virus classification or by multiple infections due to the restrictions of sample size. Since all of the above factors might influence the symptoms and clinical examinations of acute gastroenteritis, our analyses may be influenced by related bias.
